# Discovery Strategies to Maximize the Clinical Potential of T-Cell Engaging Antibodies for the Treatment of Solid Tumors

**DOI:** 10.3390/antib9040065

**Published:** 2020-11-18

**Authors:** Vladimir Voynov, Paul J. Adam, Andrew E. Nixon, Justin M. Scheer

**Affiliations:** 1 Biotherapeutics Discovery, Boehringer Ingelheim Pharmaceuticals, Inc., 900 Ridgebury Road, Ridgefield, CT 06877, USA; andrew.nixon@boehringer-ingelheim.com (A.E.N.); justin.scheer@boehringer-ingelheim.com (J.M.S.); 2 Cancer Immunology & Immune Modulation, Boehringer Ingelheim RCV GmbH & Co KG, Dr. Boehringer-Gasse 5-11, 1121 Vienna, Austria; paul.adam@boehringer-ingelheim.com

**Keywords:** T-cell engagers, bispecific antibodies, immunotherapy, oncology, antibody engineering, immunological synapse

## Abstract

T-cell Engaging bispecific antibodies (TcEs) that can re-direct cytotoxic T-cells to kill cancer cells have been validated in clinical studies. To date, the clinical success with these agents has mainly been seen in hematologic tumor indications. However, an increasing number of TcEs are currently being developed to exploit the potent mode-of-action to treat solid tumor indications, which is more challenging in terms of tumor-cell accessibility and the complexity of the tumor microenvironment (TME). Of particular interest is the potential of TcEs as an immunotherapeutic approach for the treatment of non-immunogenic (often referred to as cold) tumors that do not respond to checkpoint inhibitors such as programmed cell death protein 1 (PD-1) and programmed death ligand 1 (PD-L1) antibodies. This has led to considerable discovery efforts for, firstly, the identification of tumor selective targeting approaches that can safely re-direct cytotoxic T-cells to cancer cells, and, secondly, bispecific antibodies and their derivatives with drug-like properties that promote a potent cytolytic synapse between T-cells and tumor cells, and in the most advanced TcEs, have IgG-like pharmacokinetics for dosing convenience. Based on encouraging pre-clinical data, a growing number of TcEs against a broad range of targets, and using an array of different molecular structures have entered clinical studies for solid tumor indications, and the first clinical data is beginning to emerge. This review outlines the different approaches that have been taken to date in addressing the challenges of exploiting the TcE mode-of-action for a broad range of solid indications, as well as opportunities for future discovery potential.

## 1. Introduction

Within the last decade, therapeutic antibodies in the field of cancer immunotherapy have been used to establish a new paradigm for cancer treatment. This has mainly been driven by the clinical data and subsequent approval of several checkpoint inhibitors (CPI), and has led to more than two thousand ongoing clinical trials with these agents as monotherapy or in combination with other therapies [1]. The remarkable success of cytotoxic T-lympocyte-associated protein 4 (CTLA4), PD-1, and PD-L1 antibodies is due to their ability to antagonize immune cell checkpoint inhibitor proteins and ‘release the brake’ on the ability of a patient’s immune system to fight off tumors [2,3,4,5,6]. However, despite the high initial promise of such agents, it is now clear that only a fraction of cancer patients are showing significant clinical benefit to such agents [7]. CPI-responsive patients typically have tumors that have a high mutational burden and can be recognized by the immune system as foreign, as evidenced by the presence of tumor infiltrating lymphocytes (TILs), specifically cluster of differentiation 3 (CD3)+, CD8+ and CD4+ T-cells. Non-immunogenic tumors make up the majority of tumors across cancer indications and have no or low numbers of TILs that recognize the tumor and cannot be boosted by CPIs. For these patients, other strategies must be employed to promote the patients’ cytotoxic immune cells to recognize the tumor cells.

Two technologies have emerged that can re-direct cytotoxic T-cells, independent of their natural T-cell receptor (TCR) specificity, to tumor antigens: Chimeric Antigen Receptor T-cells (CAR-T) and T-cell Engaging bispecific antibodies (TcE). While both technologies aim to achieve a similar therapeutic effect, they are very different drug classes, with CAR-T being a cellular therapy, and TcEs protein drugs based on antibody fragments and/or soluble TCRs. Recent reviews have addressed the similarities and differences between CAR-T and T-cell Engagers [8,9]. The therapeutic approach with T-cell Engagers achieved clinical success with the approval and use of Blinatumomab for treatment of relapsed and refractory acute lymphoblastic leukemia [10]. This Bispecific T-cell Engager (BiTE) is composed of two scFv domains (one targeting CD19 on malignant B-cells and the other targeting CD3 on T-cells) connected by a linker, to induce a cytolytic synapse between a T-cell and a CD19-positive tumor cell [11]. Additional BiTEs are progressing in clinical development [12,13,14,15]; however, one drawback of BiTE molecules is their fast clearance with half-life of just a few hours, so they are administered by daily intravenous infusions.

Unlike hematologic tumors where the cancer cells often manifest themselves in the blood or tissues where lymphoid or myeloid cells are present, the majority of solid tumors have a more complex microenvironment that represents a greater challenge for cancer therapies [16,17,18,19,20]. In these cases, TcEs offer a unique opportunity by recruiting cytotoxic immune cells to the solid tumor, and once the tumor cells have been lysed there is a chain reaction involving T-cell activation, proliferation, and recruitment of other immune cells into the tumor microenvironment (TME). The presence of T-cells in the tumor environment may activate checkpoint mechanisms meaning that a combination of TcE and CPI could have synergistic therapeutic potential [18,21,22].

## 2. Clinical Use

Currently, there are no approved T-cell engagers for solid tumors. Catumaxomab (EpCAMxCD3), a prototypic version of a TcE based on a mouse–rat hybrid IgG was approved in the European Union in 2009 to treat EpCAM-positive malignant ascites [23]. However, this agent was subsequently withdrawn from the market in 2017 for commercial reasons, likely driven by the fact that the high immunogenicity of the non-human antibody backbone made it useful only in the single-dose acute ascites setting. Ertumaxomab (Her2xCD3) is another similar mouse–rat hybrid IgG bispecific molecule that was the subject of several clinical trials, but did not reach approval, again possibly due to immunogenicity, and the presence of the tumor target in normal tissues representing a toxicity risk.

**Figure 1:**
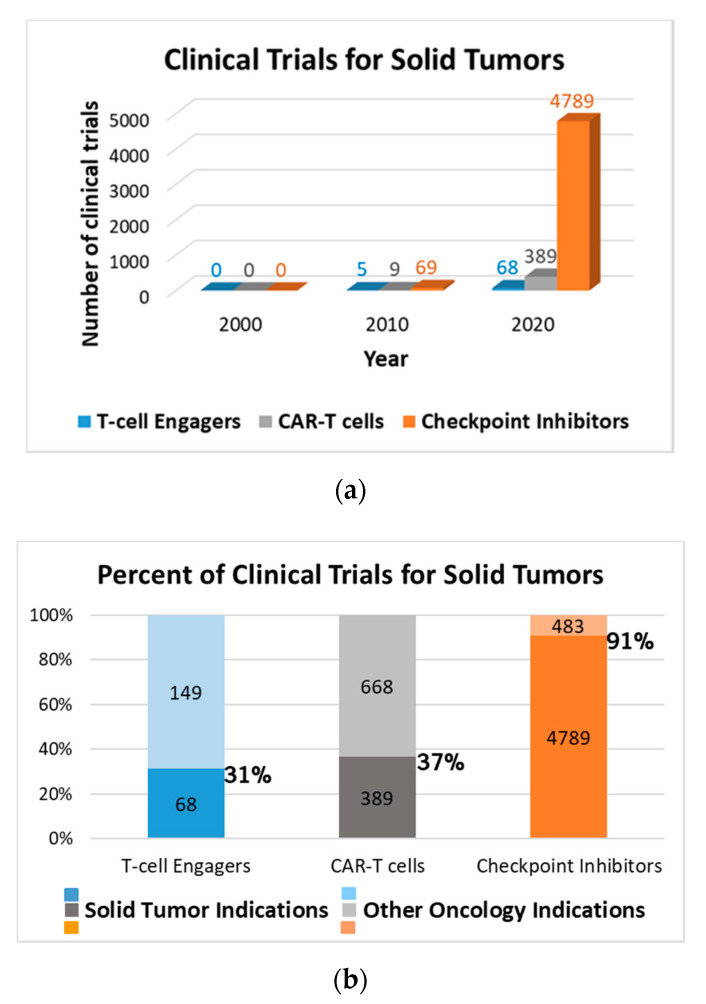
Clinical trials for solid tumors: (**a**) Comparison of number of clinical trials with TcEs, CAR-Ts and CPIs for solid tumors over time (2000, 2010, 2020). The numbers are from a search in the Citeline database by: (1) Mechanism of action: “CD3 agonism” for TcEs and “immune Checkpoint Inhibitors” for CPIs; (2) Therapeutic area: in both cases, “Oncology”, and as sub-categories all listed solid tumor indications; (3) Therapeutic modality: monoclonal antibodies and all similar classes (chimeric, humanized, human); (4) Timeframe: before year 2000, between years 2000 and 2010, and in year 2020; (**b**) Percent of clinical trials for solid tumor indications vs. all oncology indications for each of the three therapeutic modalities. The numbers inside the columns correspond to the number of clinical trials. The percent number on the side of each column indicates the percent of clinical trials for solid tumor indications vs. for all oncology indications with each of these therapeutic modalities.

Despite the fact that there is a growing number of clinical trials for TcE biotherapeutics targeting solid tumor indications, these are significantly fewer than with CAR-Ts, and far less than with CPIs (Figure 1a). Approximately a third of the clinical trials ongoing with TcEs are targeting solid tumor indications, which is similar to the ratio with CAR-Ts, while most of CPIs are towards solid tumors (Figure 1b).

These lower numbers for TcEs towards solid tumors likely reflect the higher complexity and development time of the biotherapeutic modality. Two of the most advanced TcEs targeting solid tumors with ongoing clinical trials are IMCgp100 (Tebentafusp) and RO6958688 (Cibisatamab).

Tebentafusp showed partial responses and stable disease in several patients with uveal or cutaneous melanoma, and is currently in a Phase II clinical trial for metastatic uveal melanoma [24] (Table 1). This bispecific ImmTAC molecule comprises an affinity-optimized T-cell Receptor (TCR) domain that recognizes Human Leukocyte Antigen (HLA)-gp100peptide complex on tumor cells, and an anti-CD3 scFv that binds CD3 on T-cells to re-direct and activate the T-cells to lyse the gp100 positive tumor cells. One of the on-going clinical trials, NCT02535078, aims to evaluate the efficacy of Tebentafusp in combination with anti-PD-L1 and/or anti-CTLA-4 CPIs. Several other ImmTAC TcE molecules against targets presented in complex with HLA (NY-ESO-1, LAGE-1A, MAGE-A4, PRAME) are entering clinical trials (Table 1). These studies will not only test novel TcE technologies, but also broaden the therapeutic concept of targeting tumor-specific peptides in complex with HLA.

Cibisatamab is being evaluated in several clinical trials for treatment of patients with CEA-positive solid tumors, such as non-small cell lung cancer and colorectal cancer (Table 1). The molecule has three binding domains, two Fabs that contact the target and one Fab that binds to CD3. This 2 + 1 structure allows for avid binding to the tumor antigen for improved therapeutic window, while concomitantly engaging CD3 on T-cells [25]. The molecule features a Crossmab technology for correct light-to-heavy chain pairing in the Fabs, and knob-in-hole technology for heterodimerization of the Fc. One of the clinical trials, NCT03866239, is evaluating Cibisatamab in combination with anti-PD-L1 therapy, consistent with the premise of synergies between the TcE and CPI mechanisms of action. This clinical trial also includes pre-treatment of patients with obinutuzumab (anti-CD20) to prevent occurrence of anti-drug antibodies, observed in earlier studies.

Tens of other bispecific TcEs for solid tumors are in clinical trials (Table 1). Several molecules have not progressed, possibly because of insufficient efficacy, or of toxicities due to expression of the tumor associated antigen (TAA) also in healthy tissues, or to target-independent T-cell activation. There appears to be a trend towards half-life extended TcE modalities, and many of the clinical trials are of TcEs in combination with CPI therapy. Based on the so far limited clinical experience with TcE agents in solid tumors, how can we assure the clinical translation of the TcE programs currently in pre-clinical development? Do we have the necessary technologies, or do we need new technologies? Drug discovery strategies to maximize the potential clinical benefit of the TcE therapeutic approach for solid tumors are reviewed, discussed and proposed below.

## 3. Challenges

### 3.1. Targeting Strategies for Solid Tumors

A fundamental challenge for designing effective TcE therapies for solid tumors is the identification of tumor selective targeting antigens. The identification of such tumor associated antigens (TAAs) and lineages, and their utility for the targeting of therapeutic antibodies selectively to tumors, have been an area of intense research for many years [26,27]. Several of the TcE molecules in clinical trials use TAAs, such as Her2, that have been successfully targeted in the past with other therapeutic modalities such as regular IgGs or antibody-drug conjugates. A TcE modality offers a differentiated and arguably more efficacious approach. Compared to a regular IgG, T-cell redirected cytotoxicity is considered a more potent and efficacious approach to targeting solid tumors than Fc-mediated antibody-dependent cell-mediated cytotoxicity (ADCC). Compared with antibody drug conjugates (ADC), T-cell redirected cytotoxicity relies on the host’s immune system rather than on conjugation to cytotoxic chemical payloads, and it attacks dormant as well as actively dividing cancer cells. However, targets like Her2, EpCAM, CEA, are challenging to target with the highly potent TcE mechanism of action because of basal expression on healthy tissue, despite overexpression in tumor cells.

In more recent years, detailed analysis of the transcriptome, proteome, and metabolome of diseased versus healthy cells and tissues has been used to identify tumor-selective targeting proteins [28]. One example of a cell-surface target that has emerged as highly tumor-specific is Delta-like Ligand 3 (DLL3) [29]. Earlier, an ADC approach was used, but a Phase II clinical trial with Rovalpituzumab tesirine in DLL3-expressing small-cell lung cancer did not show significant overall benefit [30]. More recently, a TcE approach was pursued towards DLL3-positive small-cell lung cancer [31,32], and the next few years will indicate whether the TcE modality confers good translation of very promising pre-clinical results into the clinic.

In another example, a recent peptidome study using mass spectrometry analysis identified HLA-complexed peptides and developed a predictive tool for neo-antigens [33]. This and other studies have led to innovative TcE approaches targeting tumor selective major histocompatibility complex (MHC)/peptide complexes [34]. The advantage of targeting tumor presented peptide antigens is that it opens up the tumor selective protein space to include intracellular proteins that could not normally be targeted with an antibody approach. Tebentafusp is the leading TcE example that uses this target class, and others have now started clinical development.

To date, the most successful approach to achieve tumor cell target-dependent activation of T-cells is via the targeting of unique epitopes on the T-cell receptor, CD3. The CD3 targeting arm of current TcEs is often derived from one of two binders identified in the 1970–80′s that bind to the CD3ε subunit, OKT3 [35] and SP34 [36,37]. Since then, different sequence optimized versions of these mouse-derived antibodies have been generated, for example to reduce the risk of immunogenicity in patients. The current thinking on CD3 affinity is that binding to CD3 with nM range affinities is advantageous over pM binding, with the expectation that weaker binding to CD3 would be less likely to cause TAA-independent T-cell activation and lysis. More recently, a screening approach was used to identify new, improved CD3 binders [38]—when formatted into a bispecific, some of these binders against different CD3 epitopes confer strong tumor cell killing, with minimal cytokine release [38], which is considered important towards maximizing a therapeutic window in the clinic. Regarding valency to CD3, having only one anti-CD3 binding arm in the multispecific TcE molecule is thought to result in a better safety profile because of lower risk of off-tumor T-cell activation. However, among all the different TcE programs that are pursued currently, there are also examples of molecules that are bivalent for CD3. In the next few years, results on the different formats and binders may give a clearer picture on the optimal options of affinities, valency, and formats.

There is also a clinical example of a TcE that uses CD28 instead of CD3 as the activating target receptor on T-cells (PSMAxCD28, NCT03972657). In addition, a recent preclinical study of co-activating CD3 and CD28 with a trispecific molecule targeting CD38 has shown significantly more potency in in vitro assays than the corresponding bispecific constructs that activate either CD3 or CD28 [39]. However, a control trispecific molecule without a CD38 binding arm also shows very strong activity, indicating that target dependent activation has been compromised. This illustrates the fine balance between potency and specificity that TcEs need to strike for optimal therapeutic benefit.

Beyond the identification of suitable tumor targeting antigens to enable TcEs, pre-clinical pharmacological analysis includes the evaluation of TcEs in in vitro potency assays and in vivo efficacy models of the disease to identify the most promising therapeutic candidates. Elegant cell-based functional assays allow evaluation of multiple target binders and formats for T-cell activation and in vitro target cell lysis [12,21,40,41,42,43,44]. Patient-derived organoid assays also help bridge cell-based functional assays with pre-clinical in vivo efficacy studies and clinical trials, as described for CEA-positive solid tumors [45]. Similarly, the establishment of elaborate in vivo models facilitate the pre-clinical evaluation and ranking of candidate therapeutics. One successfully used disease-relevant model for TcEs being developed for the treatment of solid tumors uses so called ‘immune avatar models’ which consist of immune-deficient mice to establish a human tumor xenograft, followed by engraftment of human T-cells and then administration of the TcE molecule [12,29]. Other options for models include the use of transgenic mice that express human CD3ε, substituting the need to use immune-deficient mice and to engraft human T-cells [21], although one challenge for these models is that the human target has to be introduced into the mouse tumor cells if the target binder is not mouse cross-reactive. Ueda et al. [46] constructed a human CD3 transgenic mouse with all three CD3 subunits (CD3ε, CD3δ, CD3γ) replaced, and such in vivo model was applied to the evaluation of a Glypican3 (GPC3)/CD3 TcE, ERY974 [47]. Alternatively, bispecific molecules that bind mouse CD3 can be evaluated in fully immune-competent mouse models. The disadvantage of using the murine immune system for such models is that the human tumors cannot be engrafted, and a syngeneic tumor has to be used meaning that a surrogate binder has often to be created to the murine antigen target. In one such approach, Benonisson et al. identified that a CD3-bispecific TcE recruits several immune cell types to the tumor microenvironment in a syngeneic mouse model of melanoma [48].

It remains difficult to capture all the heterogeneity and complexity of the TME of patients into pre-clinical models of disease. However, better understanding of the complex biology of solid tumors with respect to any cellular or extracellular matrix barriers (for example, stromal cells or collagen, hyaluronic acid and fibronectin-rich matrix) also contributes to the design of better immuno-oncology model systems and therapies [49]. The increased knowledge of cell types, extracellular matrix components and their interplay in the TME permits the design of well-controlled experiments that ultimately can be more predictive of positive outcome in patients. In the next few years, we will learn how well the promising pre-clinical results on several TcE programs translate in the clinic.

### 3.2. Identifying Optimal Target Binders

Usually, the next step in the biologics discovery process following the identification and validation of suitable targets is the generation of antibody binders that meet several requirements. Sophisticated discovery platforms of synthetic libraries or humanized animals add to the more traditional immunization campaigns for antibody generation. Increased structural and computational capabilities also facilitate the identification of diverse set of binders, even to challenging molecular targets or epitopes. As with any other therapeutic target or modality, binding affinity is a very important criterion. In the case of TcEs, another key parameter is epitope. Species cross-reactivity and biophysical properties further define the evaluation of binders to identify the optimal ones for TcEs.

Affinity is one important consideration for potent TcEs. Strong binding (K_D_ < 1 nM) to the TAA is considered a pre-requisite, especially for targets with a very low copy number on the surface of the tumor cells, and a notable example is Tebentafusp. Even though identification of strong, selective, binders to specific MHC-complexed peptides is challenging due to the nature of the complexed peptide, Tebentafusp exhibits a pM binding affinity [24]. Meanwhile, for a TAA that also has low expression levels on healthy normal tissues, it is possible to achieve a therapeutic window using avidity optimized weak binders (K_D_ 1–100 nM), where the molecule binds preferentially to higher-target expressing tumor cells, and sparing the lower-target expressing normal cells. For example, a bispecific format with bivalent Her2 binding improved the selectivity towards Her2-positive tumor cells over healthy cells through avidity [44]. Cibisatamab (CEAxCD3) is another molecule in such a 2:1 format, with two binding arms for the TAA, and one for CD3.

Epitope on the target is another key parameter for conferring strong TcE potency. A study by Bluemel et al. demonstrated that cell-membrane proximity of the epitope determines the potency of BiTEs, especially for large surface antigens such as melanoma chondroitin sulfate proteoglycan (MCSP) [40]. A similar observation was reported for efficient synapse formation with TcEs directed to a membrane-proximal epitope on another TAA, FcRH5, a B-cell lineage marker [50]. There may be other requirements for epitope selection, for example in cases where regions are bound by natural ligands, or due to sequence homology to other family members, or across other species. Ultimately, the epitope space for a given TAA might be significantly restricted; however, novel antibody generation strategies, as well as new discovery platforms, enable identification of binders to unique, previously inaccessible epitopes.

Species cross-reactivity is often another important criterion in lead identification. For example, binding cross-reactivity to the corresponding antigen in non-human primate species, such as cynomolgus monkey, enables important PK and safety studies before final molecule selection and clinical trials. Meanwhile, cross-reactivity to mouse or other species with disease-relevant models is important to be able to evaluate lead candidates in in vivo efficacy studies. If such cross-reactivity is not achieved or feasible, important in vivo parameters should be evaluated pre-clinically using a surrogate molecule and extrapolated to the lead therapeutic molecules. Human and cynomolgus CD3ε shows high sequence homology only in the first 30 amino acids, so this presents very limited epitope space to achieve human/cynomolgus species cross-reactivity, and human/mouse CD3ε sequences are even more divergent. Currently there are no known human/mouse CD3ε cross-reactive binders, and the only anti-mouseCD3 binder is 2C11 [51,52].

CMC properties (chemistry, manufacturing and controls) of the binders present an underlying objective throughout the discovery process. Even in regular IgGs, the Fab regions can have a profound effect on overall molecule stability and manufacturability, due to melting temperature, hydrophobicity and other biophysical and chemical properties of the CDRs and frameworks in the variable regions. Evaluation of the CMC properties of different binders is even more important in multispecific antibodies because of multiple variable domains, usage of fragment structures and non-IgG elements such as linkers and point variants for heterodimerization. High stability is incorporated in the design of recent Fab and non-Fab (scFv, VHH domains) synthetic libraries for antibody generation, by using some of the most common and most stable human germlines [53,54]. In addition, antibody libraries in non-Fab modalities are very useful for subsequent bispecific formatting with scFv and VHH components, to avoid any loss of binding or stability attributes during Fab to scFv or Fab to VHH engineering. There is an expanding set of biophysical techniques [55,56,57] and in silico tools [58,59,60] for developability assessment and engineering designs, many of which can be used in a high-throughput manner. Early manufacturability and biophysical evaluation during lead identification would advance more stable binders for bispecific TcE designs.

### 3.3. Multispecific Engineering Approaches

**Figure 2:**
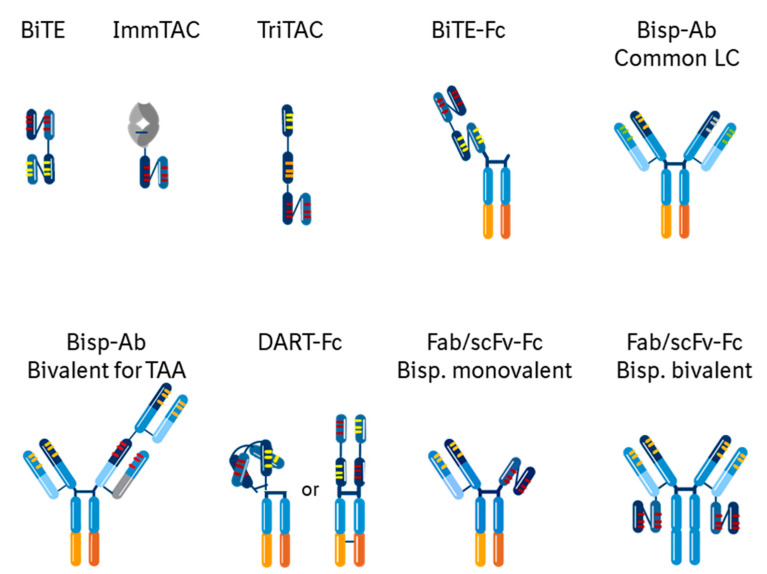
Examples of some of the Bispecific structures of T-cell Engager molecules in clinical trials. CDRs in the variable light and variable heavy chains for the same target are in the same color. Knob-in-Hole and other CH3 engineering technologies in the CH3 domain of the Fc are indicated by the different shade of orange.

One of the drawbacks to the pioneering BiTE technology was the need for continuous intravenous infusion. BiTEs proved that TcEs can be developed and commercialized. The challenge was to improve on the drug-like properties, and this has been done by addressing several aspects of the technology. Early engineering approaches to improve the biophysical stability of Fvs established the use of linkers or interchain disulfide bonds [61,62], and enabled BiTE structures. Meanwhile, Knob-in-hole mutations in the CH3 domain of the Fc were ingeniously designed to form Fc heterodimers [63], and introduced Fc-containing, more antibody-like bispecifics. Subsequent to BiTEs, further protein engineering has led to various multispecific structures such as CrossMabs with CH1-CL crossover [64], TandAbs (Tandem diabodies) [65], DARTs (dual-affinity re-targeting) [66], ITEs (IgG-like T-cell Engaging bispecific antibody) [31], BEATs (Bispecific Engagement by Antibodies based on the T cell receptor), ImmTACs (Immune mobilising monoclonal TCRs against cancer) [34], TriTACs (Tri-specific T-cell Activating Construct) [67], and numerous other small domain or full antibody-like constructs [68,69]. Several such multispecific structures are currently used in TcE modality (Figure 2, Table 1). This variety of formats permits the identification of the most potent, safe and manufacturable ones for a given therapeutic concept [25,65,70,71,72,73]: monovalent vs. bivalent binding for one or both targets, different affinity and epitope binders, different size, distance and geometry of the binding domains and the whole molecule.

Another example of important protein engineering for TcEs in light of the biology of solid tumors is the design of TME conditionally active molecules. Three TME conditions that have been explored so far are presence of specific metaloproteases, increased levels of ATP, or acidic pH. Probody is one of the first examples of a pro-drug antibody for improved therapeutic window [74]. A Probody has the antibody binding regions masked with a peptide which is processed by TME-specific proteases to then allow binding of the antibody to its target. At least four antibodies using Probody technology are in clinical trials as immunotherapy for solid tumors and also certain lymphomas [75]. The Probody concept was more recently applied to an EGFRxCD3 TcE, and preclinical studies indicate a more than 60-fold increase of maximum tolerated dose compared to unmasked bispecific construct [76]. Regarding ATP-dependent effects, Switch Antibody technology confers binding to a TAA, only in the presence of ATP, in the TME, as demonstrated for an anti-CD137 antibody [77]—the technology improves the safety profile for this target, and can be applied to other targets and platforms, including T-cell Redirecting Antibodies. Meanwhile, BioAlta describes low-pH specific binding to CD3 as part of their TcE and CAR-T platform for no off-tumor T-cell activation. All these examples of conditionally active TcEs are particularly relevant for TAAs that exhibit some basal expression in healthy tissues, and can be applied with respect to either the TAA or the T-cell antigen. Such technological advances can make more targets available and specifically help expand the usefulness of TcEs in solid tumors.

Safety considerations guide the design and characterization of TcE molecules in several additional aspects. The affinity and valency of the CD3 binder is important to assure that there is no target-independent T-cell activation, which in its worst manifestation could lead to cytokine release syndrome, and there are well-established in vitro T-cell activation assays for early screening of molecules. In addition, use of engineered Fc variants with weaker or no binding to Fcγ receptors results in significantly reduced effector function, and avoids potential undesirable cross-linking interactions of different immune cells [78,79]. Because of the presence of non-native elements such as linkers, non-Fab binding domains, swapped domains, and point variants in bispecific formats, the risk of immunogenicity of TcE molecules is higher than for regular IgGs. The multifaceted relationship of immunogenicity to sequence, stability of the molecule, antibody–target complex and higher-order structures, requires monitoring of immunogenicity and potential occurrence of anti-drug antibodies at all stages of pre-clinical and clinical investigation.

Regarding potency, several at least perceived challenges of TcEs for solid tumor indications include tumor penetration and efficiency in forming a strong immune synapse. Super-resolution and fluorescence microscopy allow a better understanding of the requirements for formation of a strong immune synapse for T-cell activation [80,81]. Meanwhile, a study with natural killer cells demonstrates that dextrans less than 4 nm are not limited in entering/exiting the immune synapse, while molecules around 10–13 nm are more than 50% impeded and dextrans greater than 32 nm are completely blocked [82]. Regular antibodies with about 150 kDa molecular weight have a hydrodynamic radius of 5–6 nm. It could be expected that smaller multispecific formats (BiTEs, ImmTACs and TriTACs are about 50 kDa) are more efficient than larger formats (about 150–200 kDa). However, based on pre-clinical in vivo studies on various TAAs, TcEs of both BiTE and bulkier HLE formats are able to achieve tumor growth inhibition and even regression with very low doses [21,25,31,41,42]. The size of the current bispecific TcE molecules does not seem to be an impediment to entering and forming a strong immune synapse.

Perhaps one of the biggest challenges for the development of bispecific antibody modalities has been the ability to ensure commercial manufacturing. In addition to identifying binders that have variable regions with good CMC properties, it is important that the complete bispecific TcE molecules are manufacturable and stable. There are a large number of multispecific formats currently available [68,71], and each of these has a unique set of manufacturability challenges, beyond platform processes for regular IgG molecules. Smaller formats such as BiTEs and ImmTACs do not allow Protein A-based affinity purification and do not benefit from molecule-stabilizing effects of antibody constant domains. On the other hand, larger biologics formats usually have an Fc domain that enables Protein A affinity purification, and confers additional stability and longer half-life. However, more often than not, TcE molecules with an Fc domain are asymmetric molecules, with different heavy and/or light chains. Expression and purification of such bispecific molecules is more challenging than that of regular IgG molecules, usually with lower expression level, lower initial purity after Protein A purification, and more polishing steps. So additional resources and time are required to build multispecific molecules with favorable CMC properties and to establish a robust manufacturing process for novel formats.

From the perspective of pharmacokinetics, a desired improvement in next-generation TcEs is the half-life of the therapeutic molecule, to allow better dosing convenience for patients. Because of their small size and domain composition (two scFvs connected by a linker), BiTE molecules clear very fast in vivo. With a short half-life of just a few hours, BiTE molecules have to be administered by continuous intravenous infusion. Several technologies, including BiTE-Fc fusions, IgG-like Fc containing formats, HSA/ABD fusion constructs, or PEGylation [83,84], enable longer half-life from at least several days to more than a week, and approaching the half-life of regular IgGs. As a result, in pre-clinical models of disease, high potency and efficacy can be achieved with once-weekly dosing over several weeks. Similarly, in the clinic, such HLE-TcEs are administered once weekly or less frequently as opposed to continuous infusion. While the PK profile of a biological drug is greatly improved by the presence of an Fc or an HSA-binding domain, the variable regions (especially if non-Fab format such as scFv or VHH) in a multispecific can significantly influence the in vivo stability and half-life of a molecule. In vitro serum stability and in vivo mouse PK studies can provide early information about which variable regions, linkers and formats are most suitable to advance for further testing.

## 4. Proposed Discovery Strategies and Conclusions

**Figure 3:**
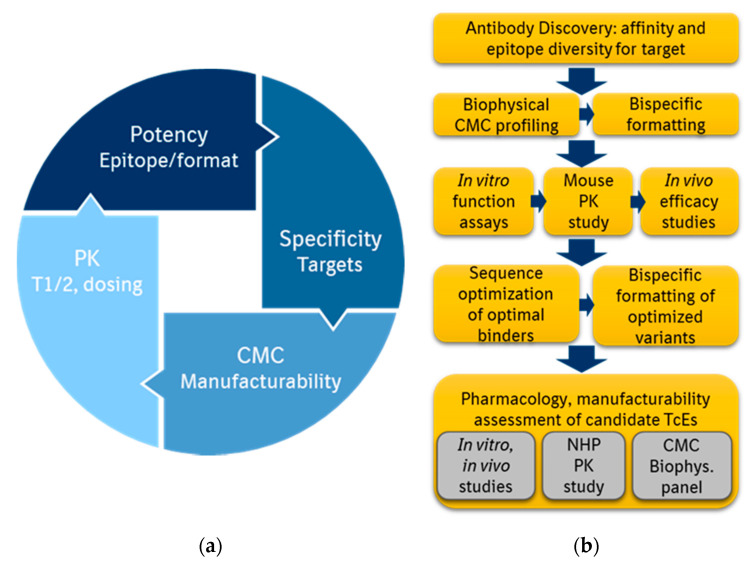
T-cell Engagers in drug discovery: (**a**) Optimal features in a half-life extended Next-Gen T-cell Engager. First generation TcEs such as BiTEs show very high potency, but very short half-life, and challenging manufacturability. Current half-life extended TcEs aim to show strong potency, similar to BiTEs, while also featuring manufacturability and PK properties similar to regular IgG biologics; (**b**) Proposed workflow for drug discovery of T-cell Engagers. Discovery of antibodies with diverse affinity and epitopes to the target is beneficial, especially of novel targets. Side-by-side function and stability profiling are recommended before and after sequence-optimization, because of the interdependent importance of both Pharmacology and CMC for the clinical success of a TcE.

The complex and heterogeneous micro-environment of solid tumors means that for many cancer types, there remains a high unmet need for effective therapies. The clinical potential of the TcE mode-of-action has been demonstrated, and this is why it is important to continue to advance efforts in discovery and manufacturing to bring new generations of TcEs to patients, and address this high unmet medical need. Next generation TcEs should strike a balance of potency, safety, manufacturability and pharmacokinetics (Figure 3a). High potency depends a lot on the affinity of the binder, epitope, and the bispecific structure, in addition to target copy number on the target cells. Specificity and safety of the disease target as well as the therapeutic candidate are closely related, while multispecific engineering can offer additional target space even for targets with low expression on normal cells but significantly higher expression on tumor cells. Due to the more complex structure of bispecific molecules than regular antibodies, TcEs pose unique CMC challenges that have to be tackled to assure good manufacturability. Regarding pharmacokinetics, most of the current TcEs in clinical trials have a half-life extended profile allowing less frequent dosing, similar to that of regular IgGs.

Expertise from different disciplines addressing these key considerations provides a roadmap for a discovery strategy to benefit identification and evaluation of TcEs for solid tumors (Table 2), from biological understanding of the therapeutic challenges and opportunities of the TME, to the identification and validation of suitable TAAs, followed by the discovery of diverse set of binders, to be then incorporated in bispecific TcE molecules, for evaluation for function, safety, manufacturability and pharmacokinetics.

We propose a workflow of TcE discovery that includes parallel considerations of function, CMC properties, safety and PK profiling (Figure 3b). Improved technologies and capabilities permit the identification of diverse sets of affinity and epitope binders for a TAA, even for very challenging cell surface targets. Biophysical characterization of the binders alone and also in the context of bispecific structures identifies the molecules to advance for in vitro function assays, followed by mouse PK and in vivo efficacy studies. Mouse PK studies help determine the half-life and in vivo stability of the bispecific molecules, and can identify any challenges due to the targets, if the binders are cross-reactive, or due to variable region or engineered element sequences. Sequence optimization of the preferred binders and bispecific molecules is done to make the sequences as human as possible and to reduce critical quality attributes such as deamidation, aspartate isomerization, oxidation or fragmentation. The optimized lead candidates are subjected to a panel of rigorous pre-clinical testing for: (1) potency in in vitro and in vivo models of disease; (2) safety, half-life and in vivo stability in non-human primates; and (3) CMC properties. Favorable outcome of such comprehensive evaluation of TcE candidate therapeutics with good manufacturability and developability properties would assure a faster path to the clinic and more efficient approval and delivery to patients.

Clinical and pre-clinical TcEs for solid tumors are against many different TAAs, and are built in various bispecific modalities. Each new entity brings a set of target-dependent and molecule-dependent challenges. Learnings from prior programs and advancements in biomedical research and development offer ways to address these challenges. The next few years promise to be of critical value in identifying optimal Immuno-oncology treatment options for solid tumor indications, where TcEs can make a unique and significant contribution, alone or in combination therapy.

## Figures and Tables

**Table 1:** Bispecific technologies and examples of specific T-cell Engager molecules in clinical trials for solid tumors. Features are derived from literature as described in the main text. Information on molecules and clinical trials is from clinicaltrials.gov and from Citeline. Of the 68 clinical trials in Figure 1, the molecules included in this table have clinical trial numbers assigned. The bispecific antibodies catumaxomab and ertumaxomab, for which all clinical trials are closed, are not included.

Technology and Key Features	Examples (Targets): Phase, Indication, Trial Number, Status (Other Information)
**BiTE:** -Two tandem scFvs; -short half-life (hours)	* **AMG 110/MT-110/solitomab (EpCAMxCD3):** -PhI, Solid tumors, NCT00635596, Completed * **MEDI-565/AMG 211/MT-111 (CEAxCD3):** -PhI, Gastrointestinal Adenocarcinomas, NCT01284231, Completed; -PhI, Advanced Gastrointestinal Cancer, NCT02291614, Completed (Terminated) * **Pasotuxizumab/AMG 212/MT-112/BAY 2010112 (PSMAxCD3):** -PhI, Prostate Cancer, NCT01723475, Completed (Terminated) * **AMG 596 (EGFRvIIIxCD3):** -PhI, Glioblastoma, NCT03296696, Recruiting (alone or in combination with AMG 404 (anti-PD-1))
**ImmTAC:** -Bispecific of a TCR domain and anti-CD3 scFv; -short half-life (hours)	* **Tebentafusp/IMCgp100 (gp100xCD3):** -Early PhI, Advanced Melanoma, NCT01209676, Completed; -PhI, Malignant Melanoma, NCT01211262, Completed; -PhII, Malignant Melanoma, NCT02889861, Terminated; -PhI/II, Malignant Melanoma, NCT02535078, Recruiting (combination with Durvalumab (anti-PD-L1) and/or Tremelimumab (anti-CTLA-4)); -PhI/II, Uveal Melanoma, NCT02570308, Active; -PhII, Uveal Melanoma, NCT03070392, Recruiting; * **IMCnieso (NY-ESO-1- and/or LAGE-1AxCD3):** -PhI/II, Advanced Solid Tumors, NCT03515551, Recruiting * **IMC-C103C (MAGE-A4xCD3):** -PhI/II, Advanced Solid Tumors, NCT03973333, Recruiting (alone and in combination with Atezolizumab (anti-PD-L1)) * **IMC-F106C (PRAMExCD3):** -PhI/II, Advanced Solid Tumors, NCT04262466, Recruiting (alone and in combination with CPIs)
**TriTAC:** -Trispecific construct: TAA-HSA-CD3, with anti-HSA binder for half-life extension	* **HPN424 (PSMAxCD3):** -PhI, Advanced Prostate Cancer, NCT03577028, Recruiting * **HPN536 (MesothelinxCD3):** -PhI/II, Advanced Cancers, NCT03872206, Recruiting
**BiTE with Fc:** -Two tandem scFvs linked to an Fc domain for half-life extension to several days, for less frequent dosing	* **AMG 160 (PSMAxCD3):** -PhI, Prostate Cancer, NCT03792841, Recruiting * **AMG 199 (MUC17xCD3):** -PhI, Gastric and Gastroesophageal Junction Cancers, NCT04117958, Recruiting * **AMG 757 (DLL3xCD3):** -PhI, Small Cell Lung Cancer, NCT03319940, Recruiting
**Bispecific Antibody with common Light Chain:** -Fab domain binders -common light chain -Fc domain for half-life extension -Fc mutations for heterodimerization of heavy chains	* **ERY974 (GPC3xCD3):** -PhI, Advanced Solid Tumors, NCT02748837, Completed; -PhI, JapicCTI-194805, Recruiting * **REGN4018 (MUC16xCD3):** -PhI/II, Recurrent Ovarian Cancer, NCT03564340, Recruiting (alone or in combination with Cemiplimab (anti-PD-1)
**DuoBody Bispecific Antibody:** -Fab domain binders -Fc domain for half-life extension -mutations in Fc -process for bispecific antibody generation from two regular IgGs after purification	* **JNJ-63898081 (PSMAxCD3):** -PhI, Advanced Stage Solid Tumors, NCT03926013, Recruiting
**Bispecific TcE with Fc and bivalent for TAA:** -Fc domain for half-life extension; -Knob-in-Hole technology in Fc for heterodimerization -CrossMab technology for correct LC-HC pairing in a bispecific -Two sites to bind TAA for improved therapeutic window.	* **Cibisatamab/RO6958688/RG7802 (CEAxCD3):** -PhI, Solid Tumors, NCT02324257, Completed; -PhI, Advanced Solid Tumors, JapicCTI-173764, Completed; -PhI, Solid Tumors, NCT02650713, Completed (in combination with Atezolizumab (anti-PD-L1)); -PhI/II, Non-small Cell Lung Cancer, NCT03337698, Recruiting; -PhI, Colorectal Cancer, NCT03866239, Active (in combination with Atezolizumab (anti-PD-L1) after pretreatment with Obinutuzumab (anti-CD20))
**DART-Fc:** -Fab or Fv domain binders with linkers -Fc domain for half-life extension -Monovalent or bivalent for targets	* **PF-06671008 (CDH3xCD3):** -PhI, Advanced Solid tumors, NCT02659631, Terminated * **MGD007 (gpA33xCD3):** -PhI, Colorectal Cancer, NCT02248805, Completed; -PhI/II, Metastatic Colorectal Cancer, NCT03531632, Active (in combination with MGA012 (anti-PD-1)) * **MGD009 (B7-H3xCD3):** -PhI, Solid Tumors, NCT02628535, Terminated; -PhI, Solid Tumors, NCT03406949, Recruiting (in combination with MGA012 (anti-PD-1)) * **PF07062119 (GUCY2CxCD3):** -PhI, Advanced or Metastatic Gastrointestinal Tumors, NCT04171141, Recruiting
**Fab/scFv-Fc Bispecific monovalent (XmAb):** -one binder is Fab; the other is scFv -Fc domain for half-life extension -engineered CH3 domain for heterodimerization	* **Tidutamab/XmAb18087 (SSTR2xCD3):** -PhI, Neuroendocrine and Gastrointestinal Stromal Tumors, NCT03411915, Recruiting * **GBR 1302/ISB 1302 (HER2xCD3):** -PhI, HER2+ Solid Tumors, NCT02829372, Terminated -PhI/II, Breast Cancer, NCT03983395, Recruiting * **AMG 509 (STEAP1xCD3):** -PhI, Prostate Cancer, NCT04221542, Recruiting * **M701 (EpCAMxCD3):** -PhI, Ascites, Solid Tumors, ChiCTR1900024144, Recruiting * **M802 (HER2xCD3):** -PhI, HER2+ Solid Tumors, ChiCTR1900024128, Recruiting
**scFv-Fc-scFv bispecific bivalent:** -scFv domain binders -Fc domain for half-life extension -Bispecific and bivalent for targets	* **ES414/APVO414/MOR209 (PSMAxCD3):** -PhI, Prostate Cancer, NCT02262910, Completed (Terminated)
**Fab/scFv-Fc bispecific bivalent:** -scFv for CD3 attached to the C-terminus of the light chain of IgG -Fc domain for half-life extension	* **Hu3F8-BsAb (GD2xCD3):** -PhI/II, Neuroblastoma, Osteosarcoma, Other Solid Tumors, NCT03860207, Recruiting;
**Other**	* **BTRC4017A/RG6194 (Her2xCD3):** -PhI, HER2+ Solid Tumors, NCT03448042, Recruiting * **GEM3PSCA (PSCAxCD3):** -PhI, Solid Tumors, NCT03927573, Recruiting * **REGN5678 (PSMAxCD28):** -PhI, Prostate Cancer, NCT03972657, Recruiting (in combination with Cemiplimab (anti-PD-1)) * **CCW702/ABBV-154 (PSMAxCD3):** -PhI, Prostate Cancer, NCT04077021, Recruiting * **AMV564 (CD33xCD3):** -PhI, Advanced Solid Tumors, NCT04128423, Recruiting * **A-337 (EpCAMxCD3):** -PhI, Advanced Solid Tumors, ACTRN12617001181392, Terminated

**Table 2:** Discovery strategy to benefit identification and evaluation of TcEs for solid tumors.

Subject	Key Considerations
TME Biology	Effectiveness of TcE modality for solid tumors Biomarkers and functional requirements of therapeutic molecule
Target Identification	Uniqueness of target for a therapeutic concept Expression profile in tumor vs. healthy cells and tissues
Lead Identification	Fab vs. non-Fab platforms for discovery of diverse set of binders Epitope, affinity, cross-reactivity, biophysical stability requirements
Multispecific formatting	Format that enables desired potency, safety, manufacturability and PK Evaluation of different binders in format for both function and CMC
CMC properties	Inherent molecule stability for optimal potency and safety Good manufacturability and developability for fast path to the clinic

## Abbreviations

ABD: Albumin binding domain; ADCC: antibody-dependent cell-mediated cytotoxicity; ADC: antibody-drug conjugate; ALL: acute lymphoblastic leukemia; BiTE: Bispecific T-cell Engager; CAR-T: chimeric antigen receptor T-cells; CMC: Chemistry, manufacturing and controls; CPI: checkpoint inhibitor; HLA: Human Leukocyte Antigen; HLE: Half-life extended (for molecule PK); HSA: Human serum albumin; MHC: major histocompatibility complex; PK: Pharmacokinetics; TAA: tumor associated antigen; TcE: T-cell Engaging bispecific antibodies; TcR: T-cell Receptor; TILs: Tumor infiltrating lymphocytes; TME: Tumor microenvironment.

## References

[1] Tang J., Yu J.X., Hubbard-Lucey V.M., Neftelinov S.T., Hodge J.P., Lin Y. (2018). Trial watch: The clinical trial landscape for PD1/PDL1 immune checkpoint inhibitors. Nat. Rev. Drug Discov..

[2] Brown J.A., Dorfman D.M., Ma F.-R., Sullivan E.L., Munoz O., Wood C.R., Greenfield E.A., Freeman G.J. (2003). Blockade of Programmed Death-1 Ligands on Dendritic Cells Enhances T Cell Activation and Cytokine Production. J. Immunol..

[3] Leach D.R., Krummel M.F., Allison J.P. (1996). Enhancement of Antitumor Immunity by CTLA-4 Blockade. Science.

[4] Nishimura H., Nose M., Hiai H., Minato N., Honjo T. (1999). Development of Lupus-like Autoimmune Diseases by Disruption of the PD-1 Gene Encoding an ITIM Motif-Carrying Immunoreceptor. Immunology.

[5] Hodi F.S., O’Day S.J., McDermott D.F., Weber R.W., Sosman J.A., Haanen J.B., Gonzalez R., Robert C., Schadendorf D., Hassel J.C. (2010). Improved Survival with Ipilimumab in Patients with Metastatic Melanoma. N. Engl. J. Med..

[6] Topalian S.L., Hodi F.S., Brahmer J.R., Gettinger S.N., Smith D.C., McDermott D.F., Powderly J.D., Carvajal R.D., Sosman J.A., Atkins M.B. (2012). Safety, Activity, and Immune Correlates of Anti–PD-1 Antibody in Cancer. N. Engl. J. Med..

[7] Haslam A., Prasad V. (2019). Estimation of the Percentage of US Patients with Cancer Who Are Eligible for and Respond to Checkpoint Inhibitor Immunotherapy Drugs. JAMA Netw. Open.

[8] Slaney C.Y., Wang P., Darcy P.K.P., Kershaw M.H. (2018). CARs versus BiTEs: A Comparison between T Cell–Redirection Strategies for Cancer Treatment. Cancer Discov..

[9] Strohl W.R., Naso M. (2019). Bispecific T-Cell Redirection versus Chimeric Antigen Receptor (CAR)-T Cells as Approaches to Kill Cancer Cells. Antibodies.

[10] Pulte D., Vallejo J., Przepiorka D., Nie L., Farrell A.T., Goldberg K.B., McKee A.E., Pazdur R. (2018). FDA Supplemental Approval: Blinatumomab for Treatment of Relapsed and Refractory Precursor B-Cell Acute Lymphoblastic Leukemia. Oncologist.

[11] Nagorsen D., Baeuerle P.A. (2011). Immunomodulatory therapy of cancer with T cell-engaging BiTE antibody blinatumomab. Exp. Cell Res..

[12] Hipp S., Tai Y.-T., Blanset D., Deegen P., Wahl J., Thomas O., Rattel B., Adam P.J., Anderson K.C., Friedrich M. (2016). A novel BCMA/CD3 bispecific T-cell engager for the treatment of multiple myeloma induces selective lysis in vitro and in vivo. Leukemia.

[13] Topp M.S., Duell J., Zugmaier G., Attal M., Moreau P., Langer C., Krönke J., Facon T., Salnikov A.V., Lesley R. (2020). Anti–B-Cell Maturation Antigen BiTE Molecule AMG 420 Induces Responses in Multiple Myeloma. J. Clin. Oncol..

[14] Rosenthal M., Balana C., Van Linde M.E., Sayehli C., Fiedler W.M., Wermke M., Massard C., Ang A., Kast J., Stienen S. (2019). Novel anti-EGFRvIII bispecific T cell engager (BiTE) antibody construct in glioblastoma (GBM): Trial in progress of AMG 596 in patients with recurrent or newly diagnosed disease. J. Clin. Oncol..

[15] Hummel H.-D., Kufer P., Grüllich C., Deschler-Baier B., Chatterjee M., Goebeler M.-E., Miller K., De Santis M., Loidl W.C., Buck A. (2020). Phase I study of pasotuxizumab (AMG 212/BAY 2010112), a PSMA-targeting BiTE (Bispecific T-cell Engager) immune therapy for metastatic castration-resistant prostate cancer (mCRPC). J. Clin. Oncol..

[16] Giraldo N.A., Sanchez-Salas R., Peske J.D., Vano Y., Becht E., Petitprez F., Validire P., Ingels A., Cathelineau X., Fridman W.H. (2019). The clinical role of the TME in solid cancer. Br. J. Cancer.

[17] Angell H., Galon J. (2013). From the immune contexture to the Immunoscore: The role of prognostic and predictive immune markers in cancer. Curr. Opin. Immunol..

[18] Galon J., Bruni D. (2019). Approaches to treat immune hot, altered and cold tumours with combination immunotherapies. Nat. Rev. Drug Discov..

[19] Galon J., Costes A., Sanchez-Cabo F., Kirilovsky A., Mlecnik B., Lagorce-Pagès C., Tosolini M., Camus M., Berger A., Wind P. (2006). Type, Density, and Location of Immune Cells Within Human Colorectal Tumors Predict Clinical Outcome. Science.

[20] Whiteside T.L. (2008). The tumor microenvironment and its role in promoting tumor growth. Oncogene.

[21] Junttila T.T., Li J., Johnston J., Hristopoulos M., Clark R., Ellerman D., Wang B.-E., Li Y., Mathieu M., Li G. (2014). Antitumor Efficacy of a Bispecific Antibody That Targets HER2 and Activates T Cells. Cancer Res..

[22] Kobold S., Pantelyushin S., Rataj F., Berg J.V. (2018). Rationale for Combining Bispecific T Cell Activating Antibodies with Checkpoint Blockade for Cancer Therapy. Front. Oncol..

[23] Linke R., Klein A., Seimetz D. (2010). Catumaxomab: Clinical development and future directions. MAbs.

[24] Damato B., Dukes J., Goodall H., Carvajal R.D. (2019). Tebentafusp: T Cell Redirection for the Treatment of Metastatic Uveal Melanoma. Cancers.

[25] Bacac M., Fauti T., Sam J., Colombetti S., Weinzierl T., Ouaret D., Bodmer W.F., Lehmann S., Hofer T., Hosse R.J. (2016). A Novel Carcinoembryonic Antigen T-Cell Bispecific Antibody (CEA TCB) for the Treatment of Solid Tumors. Clin. Cancer Res..

[26] Old L.J. (1981). Cancer immunology: The search for specificity--G. H. A. Clowes Memorial lecture. Cancer Res..

[27] Schietinger A., Philip M., Schreiber K. (2008). Specificity in cancer immunotherapy. Semin. Immunol..

[28] Sengupta S., Sun S.Q., Huang K.-L., Oh C., Bailey M.H., Varghese R., Wyczalkowski M.A., Ning J., Tripathi P., McMichael J.F. (2018). Integrative omics analyses broaden treatment targets in human cancer. Genome Med..

[29] Owen D.H., Giffin M.J., Bailis J.M., Smit M.-A.D., Carbone D.P., He K. (2019). DLL3: An emerging target in small cell lung cancer. J. Hematol. Oncol..

[30] Morgensztern D., Besse B., Greillier L., Santana-Davila R., Ready N., Hann C.L., Glisson B.S., Farago A.F., Dowlati A., Rudin C.M. (2019). Efficacy and Safety of Rovalpituzumab Tesirine in Third-Line and Beyond Patients with DLL3-Expressing, Relapsed/Refractory Small-Cell Lung Cancer: Results From the Phase II TRINITY Study. Clin. Cancer Res..

[31] Hipp S., Voynov V., Drobits-Handl B., Giragossian C., Trapani F., Nixon A.E., Scheer J.M., Adam P.J. (2020). A Bispecific DLL3/CD3 IgG-like T-cell Antibody induces anti-tumor responses in Small Cell Lung Cancer. Clin. Cancer Res..

[32] Smit M.-A.D., Borghaei H., Owonikoko T.K., Hummel H.-D., Johnson M.L., Champiat S., Salgia R., Udagawa H., Boyer M.J., Govindan R. (2019). Phase 1 study of AMG 757, a half-life extended bispecific T cell engager (BiTE) antibody construct targeting DLL3, in patients with small cell lung cancer (SCLC). J. Clin. Oncol..

[33] Sarkizova S., Klaeger S., Le P.M., Li L.W., Oliveira G., Keshishian H., Hartigan C.R., Zhang W., Braun D.A., Ligon K.L. (2019). A large peptidome dataset improves HLA class I epitope prediction across most of the human population. Nat. Biotechnol..

[34] Oates J., Jakobsen K.B. (2013). ImmTACs: Novel bi-specific agents for targeted cancer therapy. Oncoimmunology.

[35] Kung P., Goldstein G., Reinherz E.L., Schlossman S.F. (1979). Monoclonal antibodies defining distinctive human T cell surface antigens. Science.

[36] Pessano S., Oettgen H., Bhan A.K., Terhorst C. (1985). The T3/T cell receptor complex: Antigenic distinction between the two 20-kd T3 (T3-delta and T3-epsilon) subunits. EMBO J..

[37] Salmerón A., Sánchez-Madrid F., Ursa M.A., Fresno M., Alarcón B. (1991). A conformational epitope expressed upon association of CD3-epsilon with either CD3-delta or CD3-gamma is the main target for recognition by anti-CD3 monoclonal antibodies. J. Immunol..

[38] Trinklein N.D., Pham D., Schellenberger U., Buelow B., Boudreau A., Choudhry P., Clarke S.C., Dang K., Harris K.E., Iyer S. (2019). Efficient tumor killing and minimal cytokine release with novel T-cell agonist bispecific antibodies. mAbs.

[39] Wu L., Seung E., Xu L., Rao E., Lord D.M., Wei R.R., Cortez-Retamozo V., Ospina B., Posternak V., Ulinski G. (2019). Trispecific antibodies enhance the therapeutic efficacy of tumor-directed T cells through T cell receptor co-stimulation. Nat. Rev. Cancer.

[40] Bluemel C., Hausmann S., Fluhr P., Sriskandarajah M., Stallcup W.B., A Baeuerle P., Kufer P. (2010). Epitope distance to the target cell membrane and antigen size determine the potency of T cell-mediated lysis by BiTE antibodies specific for a large melanoma surface antigen. Cancer Immunol. Immunother..

[41] Brischwein K., Schlereth B., Guller B., Steiger C., Wolf A., Lutterbuese R., Offner S., Locher M., Urbig T., Raum T. (2006). MT110: A novel bispecific single-chain antibody construct with high efficacy in eradicating established tumors. Mol. Immunol..

[42] Fisher T.S., Hooper A.T., Lucas J., Clark T.H., Rohner A.K., Peano B., Elliott M.W., Tsaparikos K., Wang H., Golas J. (2017). A CD3-bispecific molecule targeting P-cadherin demonstrates T cell-mediated regression of established solid tumors in mice. Cancer Immunol. Immunother..

[43] Löffler A., Gruen M., Wuchter C., Schriever F., Kufer P., Dreier T., Hanakam F., Baeuerle P.A., Bommert K., Karawajew L. (2003). Efficient elimination of chronic lymphocytic leukaemia B cells by autologous T cells with a bispecific anti-CD19/anti-CD3 single-chain antibody construct. Leukemia.

[44] Slaga D., Ellerman D., Lombana T.N., Vij R., Li J., Hristopoulos M., Clark R., Johnston J., Shelton A., Mai E. (2018). Avidity-based binding to HER2 results in selective killing of HER2-overexpressing cells by anti-HER2/CD3. Sci. Transl. Med..

[45] Gonzalez-Exposito R., Semiannikova M., Griffiths B., Khan K., Barber L.J., Woolston A., Spain G., Von Loga K., Challoner B., Patel R. (2019). CEA expression heterogeneity and plasticity confer resistance to the CEA-targeting bispecific immunotherapy antibody cibisatamab (CEA-TCB) in patient-derived colorectal cancer organoids. J. Immunother. Cancer.

[46] Ueda O., Wada N.A., Kinoshita Y., Hino H., Kakefuda M., Ito T., Fujii E., Noguchi M., Sato K., Morita M. (2017). Entire CD3epsilon, delta, and gamma humanized mouse to evaluate human CD3-mediated therapeutics. Sci. Rep..

[47] Ishiguro T., Sano Y., Komatsu S.I., Kamata-Sakurai M., Kaneko A., Kinoshita Y., Shiraiwa H., Azuma Y., Tsunenari T., Kayukawa Y. (2017). An anti-glypican 3/CD3 bispecific T cell-redirecting antibody for treatment of solid tumors. Sci. Transl. Med..

[48] Benonisson H., Altıntaş I., Sluijter M., Verploegen S., Labrijn A.F., Schuurhuis D.H., Houtkamp M.A., Verbeek J.S., Schuurman J., Van Hall T. (2018). CD3-Bispecific Antibody Therapy Turns Solid Tumors into Inflammatory Sites but Does Not Install Protective Memory. Mol. Cancer Ther..

[49] Puré E., Lo A. (2016). Can Targeting Stroma Pave the Way to Enhanced Antitumor Immunity and Immunotherapy of Solid Tumors?. Cancer Immunol. Res..

[50] Li J., Stagg N.J., Johnston J., Harris M.J., Menzies S.A., DiCara D., Clark V., Hristopoulos M., Cook R., Slaga D. (2017). Membrane-Proximal Epitope Facilitates Efficient T Cell Synapse Formation by Anti-FcRH5/CD3 and Is a Requirement for Myeloma Cell Killing. Cancer Cell.

[51] Dépis F., Hatterer E., Ballet R., Daubeuf B., Cons L., Glatt S., Reith W., Kosco-Vilbois M., Dean Y. (2013). Characterization of a surrogate murine antibody to model anti-human CD3 therapies. mAbs.

[52] Leo O., Foo M., Sachs D.H., Samelson L.E., Bluestone J.A. (1987). Identification of a monoclonal antibody specific for a murine T3 polypeptide. Proc. Natl. Acad. Sci. USA.

[53] Tiller T., Schuster I., Deppe D., Siegers K., Strohner R., Herrmann T., Berenguer M., Poujol D., Stehle J., Stark Y. (2013). A fully synthetic human Fab antibody library based on fixed VH/VL framework pairings with favorable biophysical properties. mAbs.

[54] Valadon P., Pérez-Tapia S.M., Nelson R.S., Guzmán-Bringas O.U., Arrieta-Oliva H.I., Gómez-Castellano K.M., Pohl M.A., Almagro J.C. (2019). ALTHEA Gold Libraries™: Antibody libraries for therapeutic antibody discovery. mAbs.

[55] Jain T., Sun T., Durand S., Hall A., Houston N.R., Nett J.H., Sharkey B., Bobrowicz B., Caffry I., Yu Y. (2017). Biophysical properties of the clinical-stage antibody landscape. Proc. Natl. Acad. Sci. USA.

[56] Kim D.M., Yao X., Vanam R.P., Marlow M.S. (2019). Measuring the effects of macromolecular crowding on antibody function with biolayer interferometry. mAbs.

[57] Liu Y., Caffry I., Wu J., Geng S.B., Jain T., Sun T., Reid F., Cao Y., Estep P., Yu Y. (2013). High-throughput screening for developability during early-stage antibody discovery using self-interaction nanoparticle spectroscopy. mAbs.

[58] Chennamsetty N., Voynov V., Kayser V., Helk B., Trout B.L. (2009). Design of therapeutic proteins with enhanced stability. Proc. Natl. Acad. Sci. USA.

[59] Kumar S., Plotnikov N.V., Rouse J.C., Singh S.K. (2017). Biopharmaceutical Informatics: Supporting biologic drug development via molecular modelling and informatics. J. Pharm. Pharmacol..

[60] Voynov V., Chennamsetty N., Kayser V., Helk B., Trout B.L. (2009). Predictive tools for stabilization of therapeutic proteins. mAbs.

[61] Glockshuber R., Malia M., Pfitzinger I., Plueckthun A. (1990). A comparison of strategies to stabilize immunoglobulin Fv-fragments. Biochemistry.

[62] Jung S.-H., Pastan I., Lee B. (1994). Design of interchain disulfide bonds in the framework region of the Fv fragment of the monoclonal antibody B3. Proteins Struct. Funct. Bioinform..

[63] Atwell S., Ridgway J.B., A Wells J., Carter P. (1997). Stable heterodimers from remodeling the domain interface of a homodimer using a phage display library. J. Mol. Biol..

[64] Schaefer W., Regula J.T., Bähner M., Schanzer J., Croasdale R., Dürr H., Gassner C., Georges G., Kettenberger H., Imhof-Jung S. (2011). Immunoglobulin domain crossover as a generic approach for the production of bispecific IgG antibodies. Proc. Natl. Acad. Sci. USA.

[65] McAleese F., Eser M. (2012). RECRUIT-TandAbs®: Harnessing the immune system to kill cancer cells. Futur. Oncol..

[66] Moore P.A., Shah K., Yang Y., Alderson R., Roberts P., Long V., Liu D., Li J.C., Burke S., Ciccarone V. (2018). Development of MGD007, a gpA33 x CD3-Bispecific DART Protein for T-Cell Immunotherapy of Metastatic Colorectal Cancer. Mol. Cancer Ther..

[67] Austin R., Aaron W., Baeuerle P., Barath M., Jones A., Jones S.D., Law C.-L., Kwant K., Lemon B., Muchnik A. (2018). Abstract 1781: HPN536, a T cell-engaging, mesothelin/CD3-specific TriTAC for the treatment of solid tumors. Immunology.

[68] Spiess C., Zhai Q., Carter P.J. (2015). Alternative molecular formats and therapeutic applications for bispecific antibodies. Mol. Immunol..

[69] Brinkmann U., Kontermann R.E. (2017). The making of bispecific antibodies. mAbs.

[70] Ellerman D. (2019). Bispecific T-cell engagers: Towards understanding variables influencing the in vitro potency and tumor selectivity and their modulation to enhance their efficacy and safety. Methods.

[71] Husain B., Ellerman D. (2018). Expanding the Boundaries of Biotherapeutics with Bispecific Antibodies. BioDrugs.

[72] Runcie K., Budman D.R., John V., Seetharamu N. (2018). Bi-specific and tri-specific antibodies- the next big thing in solid tumor therapeutics. Mol. Med..

[73] Shiraiwa H., Narita A., Kamata-Sakurai M., Ishiguro T., Sano Y., Hironiwa N., Tsushima T., Segawa H., Tsunenari T., Ikeda Y. (2019). Engineering a bispecific antibody with a common light chain: Identification and optimization of an anti-CD3 epsilon and anti-GPC3 bispecific antibody, ERY974. Methods.

[74] Desnoyers L.R., Vasiljeva O., Richardson J.H., Yang A., Menendez E.E.M., Liang T.W., Wong C., Bessette P.H., Kamath K., Moore S.J. (2013). Tumor-Specific Activation of an EGFR-Targeting Probody Enhances Therapeutic Index. Sci. Transl. Med..

[75] Autio K.A., Boni V., Humphrey R.W., Naing A. (2019). Probody Therapeutics: An Emerging Class of Therapies Designed to Enhance On-Target Effects with Reduced Off-Tumor Toxicity for Use in Immuno-Oncology. Clin. Cancer Res..

[76] Boustany L.M., Wong L., White C.W., Diep L., Huang Y., Liu S., Richardson J.H., Kavanaugh W.M., Irving B.A. Abstract A164: EGFR-CD3 bispecific Probody™ therapeutic induces tumor regressions and increases maximum tolerated dose > 60-fold in preclinical studies. Proceedings of the Abstracts: AACR-NCI-EORTC International Conference: Molecular Targets and Cancer Therapeutics.

[77] Kamata-Sakurai M., Narita Y., Hori Y., Nemoto T., Uchikawa R., Honda M., Hironiwa N., Taniguchi K., Shida-Kawazoe M., Metsugi S. (2020). Antibody to CD137 activated by extracellular adenosine triphosphate is tumor selective and broadly effective in vivo without systemic immune activation. Cancer Discov..

[78] Wang X., Mathieu M., Brezski R.J. (2018). IgG Fc engineering to modulate antibody effector functions. Protein Cell.

[79] Xu D., Alegre M.-L., Varga S.S., Rothermel A.L., Collins A.M., Pulito V.L., Hanna L.S., Dolan K.P., Parren P.W., Bluestone J.A. (2000). In Vitro Characterization of Five Humanized OKT3 Effector Function Variant Antibodies. Cell. Immunol..

[80] Dustin M.L., Baldari C.T. (2017). The Immune Synapse: Past, Present, and Future. Adv. Struct. Safety Stud..

[81] Huppa J.B., Gleimer M., Sumen C., Davis M.M. (2003). Continuous T cell receptor signaling required for synapse maintenance and full effector potential. Nat. Immunol..

[82] Cartwright A.N.R., Griggs J., Davis D.M. (2014). The immune synapse clears and excludes molecules above a size threshold. Nat. Commun..

[83] Müller D., Karle A., Meissburger B., Höfig I., Stork R., Kontermann R.E. (2007). Improved Pharmacokinetics of Recombinant Bispecific Antibody Molecules by Fusion to Human Serum Albumin. J. Biol. Chem..

[84] Stork R., Campigna E., Robert B., Müller D., Kontermann R.E. (2009). Biodistribution of a Bispecific Single-chain Diabody and Its Half-life Extended Derivatives. J. Biol. Chem..

